# NOD2-mediated immune dysregulation linking hidradenitis suppurativa and Yao syndrome: Insights from 2 cases

**DOI:** 10.1016/j.jdcr.2025.07.018

**Published:** 2025-08-16

**Authors:** Christeebella Akpala, Nessa Aghazadeh-Mohandesi, John M. Davis, Afsaneh Alavi

**Affiliations:** a Mayo Clinic Alix School of Medicine, Rochester, Minnesota; b Department of Dermatology, Mayo Clinic, Rochester, Minnesota; c Division of Rheumatology, Mayo Clinic, Rochester, Minnesota

**Keywords:** autoimmune, hidradenitis suppurativa, innate immunity, NOD 2, syndromic HS, YAO

## Introduction

Hidradenitis suppurativa (HS) is a debilitating, chronic inflammatory condition primarily affecting intertriginous areas, often leading to recurrent nodule, abscess and tunnel formation, with subsequent scarring.^1^ HS pathogenesis involves follicular occlusion and a dysregulated immune response with prominent involvement of cytokines such as tumor necrosis factor-α, interleukin-1β, and interleukin-17.^2^ Although often considered a localized follicular occlusion disorder, HS can also present as part of broader systemic or autoinflammatory syndromes in a subset of patients. These include well-characterized syndromes such as PASH (pyoderma gangrenosum, acne, and HS)^3^ and PAPA (pyogenic arthritis, pyoderma gangrenosum, and acne),^4^ as well as variants associated with mutations in genes such as *proline-serine-threonine phosphatase interacting protein 1* and *nicastrin*, all underscoring the strong genetic and immunologic basis of syndromic HS. Yao syndrome—a relatively recent addition to the spectrum of autoinflammatory disorders—is characterized by periodic fevers, diverse cutaneous manifestations (ranging from erythematous plaques to linear, scratch-like rashes), inflammatory arthralgia/arthritis, distal extremity swelling, sicca symptoms, and gastrointestinal symptoms.^5^^,^^6^ Although not traditionally associated with HS, its pathogenesis involves variants in Nucleotide-binding oligomerization domain containing 2 (*NOD2*), a gene also implicated in Crohn's disease, suggesting shared immunologic pathways that may link the 2 conditions. In this case series, we present 2 patients whose clinical features suggest a possible syndromic overlap between HS and Yao syndrome, raising the hypothesis of a broader, shared autoinflammatory axis not yet described in the literature.

## Case presentations

### Patient 1

A 50-year-old male with a complex medical history—including poorly controlled type 2 diabetes mellitus (HbA1c 12%), idiopathic pancreatitis, end-stage renal disease on hemodialysis, congestive heart failure, hypertension, obesity (BMI 32.5), chronic iron deficiency anemia, and a 2-pack-per-day smoking history—presented with a long-standing history of HS, classified as Hurley stage III, affecting the gluteal and perineal regions. His disease had been refractory to multiple systemic and procedural interventions, including 15 surgical procedures such as incision and drainage and wide local excisions. Previous medical therapies included doxycycline, adalimumab (discontinued due to heart failure exacerbation), and ertapenem (discontinued following the development of Clostridioides difficile colitis); infliximab was avoided given his cardiac risk. Most recently, he was initiated on secukinumab with ongoing therapy at the time of assessment. Physical examination revealed numerous interconnected sinus tracts, nodules, and hypertrophic scarring in the gluteal and perineal regions, without signs of acute infection. Laboratory tests demonstrated anemia (hemoglobin 8.5 g/dL), leukocytosis (white blood cell count 21.6 × 10^9^/L with neutrophilia), hyponatremia (sodium 130 mmol/L), renal dysfunction (creatinine 2.32 mg/dL), hyperglycemia (glucose 524 mg/dL), markedly elevated alkaline phosphatase (2453 U/L) and gamma-glutamyl transferase (1952 U/L), and a highly elevated C-reactive protein (49.5 mg/L), consistent with systemic inflammation. In addition to cutaneous symptoms, the patient endorsed chronic diarrhea and tenesmus, prompting extensive gastrointestinal workup. Diagnostic evaluations, including pelvic magnetic resonance imaging, MR enterography, and colonoscopy with biopsies, were negative for inflammatory bowel disease. Due to ongoing systemic symptoms and atypical features, genetic testing was pursued and revealed heterozygosity for the *NOD2 c.2938dup (p.Leu980ProfsTer2)*, also known as the *1007fs* variant. Based on the constellation of chronic inflammatory symptoms, dermatologic findings, and the presence of a known pathogenic *NOD2* mutation, a diagnosis of Yao syndrome was established. At the time of reporting, the patient had been receiving secukinumab for 4 months with partial improvement in both cutaneous disease and systemic symptoms, and long-term monitoring remains ongoing.

### Patient 2

A 44-year-old woman with a history of human leukocyte antigen-B27 positivity presented with a multisystem illness marked by episodic joint and spinal pain, malar and truncal rash, peripheral edema, recurrent low-grade fevers, periorbital swelling, and chronic gastrointestinal symptoms including bloating and loose stools. She had been treated with nonsteroidal anti-inflammatory drugs and methotrexate with some relief of joint symptoms, and with adalimumab (up to 80 mg weekly) for Hurley stage I–II HS, which improved skin symptoms but not her systemic complaints. Prior trials of etanercept and secukinumab were ineffective. At our evaluation, she continued to report diffuse musculoskeletal and systemic symptoms. Physical examination revealed inflamed papulonodules with postinflammatory changes on the trunk and axillae. Scattered milia cysts and mild hyperpigmentation were noted in the inframammary, groin, and buttock regions. Gastrointestinal evaluation included multiple endoscopies, one of which revealed lymphocytic colitis. Laboratory workup revealed elevated interleukin-18 (878 pg/mL), and genetic testing identified heterozygosity for the *NOD2 c.2938dup (p.Leu980ProfsTer2, or 1007fs)* variant. Given the constellation of symptoms, cutaneous and joint involvement, gastrointestinal findings, and presence of a pathogenic *NOD2* variant, a diagnosis of Yao syndrome was established. After 13 months on adalimumab, at the time Yao syndrome was diagnosed, she was switched to canakinumab, an Interleukin-1 beta blocker. Canakinumab is not Food Drug Administration approved for Yao syndrome, but multiple observational studies have reported its effectiveness in this condition. She continues canakinumab therapy and has shown marked improvement after 5 months, with ongoing long-term monitoring.

## Discussion

The *NOD2* gene encodes a cytoplasmic pattern recognition receptor critical for recognizing muramyl dipeptide, a component of bacterial cell walls. Activation of *NOD2* leads to downstream Nuclear factor kappa-light-chain-enhancer of activated B cells signaling and release of proinflammatory cytokines.^7^, ^8^, ^9^ While variants in *NOD2*, such as the *1007fs* frameshift mutation, are classically linked to Crohn's disease,^10^ heterozygous *NOD2* mutations have also been identified in patients with Yao syndrome, where they are believed to amplify systemic autoinflammatory responses.^6^^,^^11^^,^^12^ The co-occurrence of Yao syndrome and HS in our 2 patients—both harboring *NOD2* variants—suggests a potential syndromic overlap not previously described in the literature.

### Shared autoinflammatory mechanisms of HS and Yao syndrome?

HS is increasingly understood as a heterogeneous, polygenic autoinflammatory disease, particularly in its syndromic forms. Mutations in gamma-secretase complex genes (eg, *nicastrin*, *presenilin enhancer of gamma secretase subunit*, *presenilin-1*) underlie familial HS and contribute to dysregulated Notch signaling, impacting follicular structure and immune homeostasis.^13^^,^^14^ More recently, studies have explored *NOD2* as a novel contributor to inflammatory signaling in sporadic and syndromic HS.^15^ Our findings support this expanding framework, with both patients exhibiting *NOD2* variants and HS within the broader context of systemic autoinflammatory disease.

Additionally, the similarity in cytokine profiles—particularly involving tumor necrotizing factor alpha, IL-1β, and IL-17—across Yao syndrome and HS reinforces the notion of a shared inflammatory milieu. This cytokine overlap may underpin the coexistence of cutaneous lesions with systemic symptoms, further suggesting a syndromic interface and potential treatment options.

### NOD2 as hereditary predisposition for HS

While genetic susceptibility is central to syndromic HS, environmental factors likely modulate disease expression. Patient 1's long-standing HS was complicated by multiple comorbidities, including obesity and tobacco use, which are known to negatively impact disease course and treatment outcomes. These comorbidities are known to exacerbate HS through mechanisms involving mechanical stress, hormonal signaling, and local cytokine production.

In contrast, Patient 2, who was neither obese nor a smoker, exhibited a distinct inflammatory rheumatologic background, raising the possibility that underlying immune dysregulation—rather than conventional HS risk factors—may contribute to disease expression. Emerging evidence suggests that certain *NOD2* gene variants, while not pathognomonic for syndromic HS, may confer a hereditary predisposition that primes the immune system toward dysregulated inflammation. In this context, the clinical manifestation of HS may arise from the interaction of these genetic susceptibilities with environmental modifiers and systemic comorbidities, rather than from a classical monogenic syndrome. Such observations underscore the multifactorial pathogenesis of HS, where gene-environment interplay can variably influence disease phenotype.

### Therapeutic implications of HS with underlying NOD2 variants

The therapeutic trajectories of these 2 patients highlight the complexities of managing syndromic HS particularly when it overlaps with systemic autoinflammatory conditions like Yao syndrome or Crohn's. After Patient 1 failed to achieve clinical or biomarker remission with tumor necrotizing factor alpha inhibition (adalimumab) and antibiotics, we transitioned therapy to secukinumab to more directly target the patient's ongoing systemic inflammation given growing evidence implicating IL-17 in persistent autoinflammation. Although NOD2 risk alleles are strongly associated with Crohn's disease, and IL-17 blockade has been linked to new or worsening intestinal inflammation, we judged that the potential benefit outweighed the risk. We obtained baseline gastrointestinal evaluation including fecal calprotectin and gastroenterology consultation, and we monitored the patient closely for any new or worsening symptoms. Over 5 months of secukinumab therapy, no clinical or biomarker evidence of intestinal inflammation emerged.

Patient 2's case, characterized by a partial and transient response to tumor necrotizing factor alpha inhibitors and the emergence of noninflammatory HS lesions, raised clinical suspicion of a predominantly autoinflammatory mechanism. Elevated IL-18 levels further supported this phenotype, prompting consideration of targeted modulation of the IL-1 pathway. These observations highlight the potential utility of cytokine profiling to guide immunotherapeutic strategies in complex or syndromic HS. Rather than applying a uniform treatment algorithm, patients with suspected genetic or autoinflammatory contributions to HS may benefit from individualized approaches—including, in selected cases, consideration of IL-1 pathway inhibition tailored to their specific immune dysregulation.

### Pathophysiologic insights and future directions

The shared *NOD2* variants and overlapping cytokine signatures in our patients reinforce a potential unifying pathway centered around innate immune dysregulation, which bridges HS and Yao syndrome. This overlap may explain atypical presentations of HS—such as recurrent fevers, gastrointestinal symptoms, and rheumatologic involvement—that deviate from the usual disease course. Recognizing these features as part of a syndromic HS spectrum could lead to earlier diagnoses, more accurate subclassification of HS phenotypes, and biomarker-driven treatment strategies. Future research should focus on expanding our understanding of syndromic hidradenitis suppurativa through larger cohort studies investigating the prevalence of *NOD2* variants in HS populations, as well as cytokine profiling to stratify patients according to distinct immune signatures. Additionally, there is a growing need to investigate novel syndromic entities in which HS may represent a sentinel or early clinical marker of broader autoinflammatory disease. However, efforts to define such associations are complicated by the interpretive challenges surrounding certain genetic variants, particularly those in the *NOD2* gene. While *NOD2* mutations—such as *R702W, G908R, and 1007fs*—have been implicated in immune dysregulation, they are also relatively prevalent in the general population, limiting their specificity as diagnostic biomarkers. Moreover, *NOD2* variants are strongly associated with Crohn's disease, highlighting the importance of considering subclinical or coexisting inflammatory bowel disease in patients with atypical HS presentations. These overlapping genetic and clinical signals underscore the need for a cautious and context-driven interpretation of immune-genetic findings, particularly when considering HS within a syndromic or autoinflammatory framework. A multidisciplinary approach integrating dermatology, rheumatology, and gastroenterology may be critical for unraveling these complex disease intersections and tailoring more precise diagnostic and therapeutic strategies.

## Conclusion

In summary, this case series presents 2 patients with overlapping features of HS and Yao syndrome, unified by the presence of *NOD2* variants and converging inflammatory profiles. While these findings support the existence of a syndromic variant of HS within the broader category of autoinflammatory disorders, they also underscore the interpretive challenges posed by genetic data—particularly given the prevalence of *NOD2* mutations in the general population and their established link to inflammatory bowel disease. Rather than definitive markers of syndromic HS, such variants may reflect an underlying genetic susceptibility that, in combination with environmental or immune-mediated factors, contributes to disease expression. We propose that a subset of HS patients, especially those with systemic symptoms, refractory disease, or gastrointestinal comorbidity—may warrant consideration for reclassification under a broader *NOD2*-associated disease framework. Continued investigation into the clinical and molecular heterogeneity of HS, including its intersection with Yao syndrome and IBD, will be essential to advancing precision diagnosis and individualized immunomodulatory therapies for this complex and understudied patient population.

## Conflicts of interest

Dr Alavi serves as the board member of Hidradenitis Suppurativa Foundation; received grants from NIH, HSF, and La Roche-Posay; serves as the chair of Independent Data Monitoring: InflaRX, Almirall, Zura bio; and serves as a consultant for AbbVie, Almirall, Avalo, BI, Cantargia, InflaRx, Incyte, Leo, Sanofi, Navigator, Novartis, and UCB; and serves as an investigator for BI and Processa. Akpala, Aghazadeh-Mohandesi, and Davis have no conflicts of interest to declare.
